# Sustainable agriculture: leveraging microorganisms for a circular economy

**DOI:** 10.1007/s00253-024-13294-0

**Published:** 2024-08-30

**Authors:** Till Glockow, Anne-Kristin Kaster, Kersten S. Rabe, Christof M. Niemeyer

**Affiliations:** 1 Acheron GmbH, Auf Der Muggenburg 30, 28217 Bremen, Germany; 2 Karlsruhe Institute of Technology (KIT), Institute for Biological Interfaces 5 (IBG-5), Biotechnology and Microbial Genetics, Hermann-Von-Helmholtz-Platz 1, 76344 Eggenstein-Leopoldshafen, Germany; 3 Karlsruhe Institute of Technology (KIT), Institute for Biological Interfaces 1 (IBG-1), Biomolecular Micro- and Nanostructures, Hermann-Von-Helmholtz-Platz 1, 76344 Eggenstein-Leopoldshafen, Germany

**Keywords:** Indoor farming, Microbiome, Omics, Probiotics, Sequencing

## Abstract

**Abstract:**

Microorganisms serve as linchpins in agricultural systems. Classic examples include microbial composting for nutrient recovery, using microorganisms in biogas technology for agricultural waste utilization, and employing biofilters to reduce emissions from stables or improve water quality in aquaculture. This mini-review highlights the importance of microbiome analysis in understanding microbial diversity, dynamics, and functions, fostering innovations for a more sustainable agriculture. In this regard, customized microorganisms for soil improvement, replacements for harmful agrochemicals or antibiotics in animal husbandry, and (probiotic) additives in animal nutrition are already in or even beyond the testing phase for a large-scale conventional agriculture. Additionally, as climate change reduces arable land, new strategies based on closed-loop systems and controlled environment agriculture, emphasizing microbial techniques, are being developed for regional food production. These strategies aim to secure the future food supply and pave the way for a sustainable, resilient, and circular agricultural economy.

**Key points:**

• *Microbial strategies facilitate the integration of multiple trophic levels, essential for cycling carbon, nitrogen, phosphorus, and micronutrients.*

• *Exploring microorganisms in integrated biological systems is essential for developing practical agricultural solutions.*

• *Technological progress makes sustainable closed-entity re-circulation systems possible, securing resilient future food production.*

**Graphical Abstract:**

Microorganisms connect plant and animal agriculture through complex cycles involving carbon, nitrogen, phosphate, and additional micronutrients. This mini-review outlines the current and potential future roles of microorganisms in agroindustry.

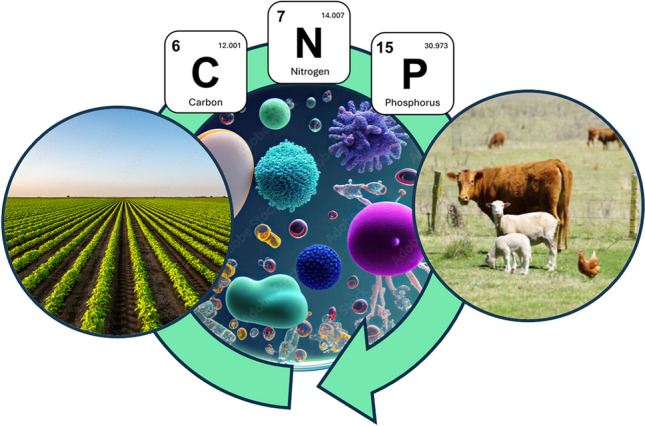

## Introduction

The role of microorganisms in agriculture has evolved significantly from past practices to present innovations. The importance of microorganisms in agriculture has been known since ancient times, as early agricultural practices such as composting relied on the often collaborative activities of bacteria, fungi, algae, protozoa, and viruses that decompose organic matter, form humus, mobilize nitrogen, phosphate, and potassium for uptake by plants and also help to control pathogens and pests (Guttmann 2005). Although a specific technical use of microorganisms for the fermentation of cereals dates back to the seventh millennium before Christ (McGovern et al. 2004), it was not until the late 19th and early twentieth centuries that scientists began to understand the profound impact of microorganisms on soil fertility, plant health, and the overall impact on the entire ecosystem of our planet (Leff et al. 2015). It is now clear that today, where climate change is affecting most life on Earth, understanding the critical role of microorganisms is crucial to mitigate and achieve environmental sustainability (Cavicchioli et al. 2019). In today’s agriculture, microorganisms already play a crucial as they impact the complex and vital carbon (Battin et al. 2009), nitrogen (Gruber and Galloway 2008) and phosphate (Oelkers et al. 2018) cycles that link plant and animal agriculture, shaping both current operations and future innovations for a sustainable circular economy. In this mini-review, we aim to highlight current trends in the use of microbial communities in plant and animal agriculture to reduce environmental impact while achieving higher yields. Against the backdrop of a growing global population and the threats posed by climate change, we will also highlight new innovations that can transform conventional agriculture by utilizing microbial feed and food production from previously unused residual and waste streams. These include alternative biological production systems such as bacteria and microalgae as well as new circular processes such as indoor farming in closed environments to increase the efficiency, resilience, and environmental sustainability of food production.

## Plant-based agriculture

Plant-based and animal-based agriculture are the fundamental sectors of global food production. The former involves the cultivation of crops such as grains, vegetables, and fruits, directly contributing to a significant portion of global food production. According to the Food and Agriculture Organization (FAO) of the United Nations, about 80% of the world’s calories come from plants, including cereals, legumes, and oilseeds. In plant-based agriculture, bacteria (e.g., *Actinomycetales*) and fungi facilitate the transformation of residuals and by-products into microbial fertilizer, meeting quality and safety standards for plant-based agriculture in a simple, rapid, safe, cost-effective, and eco-friendly manner (Mahish et al. 2024). Furthermore, microorganisms influence the functions of the ecosystem and play a crucial role in the physiology of the plant host. Therefore, agricultural and biotechnological strategies are being developed to promote microbial diversity in order to ensure long-term soil sustainability and increase plant productivity (Das et al. 2022; Nobin et al. 2023; Souza et al. 2015). A shift from harmful agrochemicals to bio-derived alternatives is underway, by recognizing the efficacy and ecological role of microorganisms. Thus, efforts are being made to enhance plant health and biomass yields by utilizing plant growth–promoting microorganisms (PGPM) and engineering microbial communities in the rhizosphere and soil. These approaches are commonly understood as “microbiome engineering,” an emerging technology that is increasingly influencing not only plant but also animal-based agriculture.

## Microbiome engineering

Soil as a complex microhabitat has two main characteristics: a large microbial diversity and a structured, heterogeneous nature with limited essential nutrients and energy sources. In the rhizosphere of higher plants, soil microflora, especially agriculturally important microorganisms such as plant growth–promoting *rhizobacteria* and fungi, *actinomycetes*, *mycorrhiza*, and endophytes, significantly influence soil and plant health through direct and indirect mechanisms, including molecular signaling (Ling et al. 2022; Trivedi et al. 2020). Plant signaling molecules are critical for root colonization, modulation of root system architecture, cell communication, gene regulation, and development of immunity, ultimately affecting plant health (Finkel et al. 2020; Khan et al. 2020; Khattab et al. 2023; Korenblum et al. 2020). In order to promote the integration of microorganisms useful to increase plant productivity, suitable bioformulations are therefore being explored in the field of “Soil Engineering” or “Rhizobium Engineering” (Hakim et al. 2021; Solanki et al. 2024; Wang and Kuzyakov 2024). The development of model-based inoculants is used for strain selection and clarification of efficacy under field conditions, possible risks for the biosphere, and finally aspects of economic implementation through product specifications and registration (Orozco-Mosqueda et al. 2022; Rai et al. 2021).

However, the interaction between microorganisms and plants is reciprocal, as uncontrollable factors such as the weather and agricultural practices influence microbial communities in specific ways through the type of land usage (e.g., plant type) and pollution sources (e.g., fertilizers), which alter the composition and function of microbial communities, and thus the natural cycles of carbon, nitrogen, and phosphorus transformation (Cavicchioli et al. 2019). Indeed, the integration of soil microbial carbon cycling and its drivers is essential for accurate modeling of biogeochemical cycles and effectively addressing the challenges of global climate change (Wu et al. 2024). Therefore, deciphering the extensive functionality and structural diversity of the plant–soil microbiome is imperative to effectively utilize these organisms in sustainable agriculture (Jing et al. 2022; Zhao et al. 2021).

Although both the plant and soil microbiome have been studied for decades, the efficiency of translating laboratory and greenhouse results to the field largely depends on the ability of beneficial microorganisms to colonize the soil and maintain ecosystem stability. In addition, the plant and its biotic and abiotic environment are variables that influence the diversity and structure of the microbiome of plants and soils. In recent years, researchers have been looking into “Microbiome Engineering,” which allows them to modify microbial communities to increase the efficiency and effectiveness of inoculants (Afridi et al. 2022; Nadarajah and Abdul Rahman 2023). Modern “omics” (genomics, transcriptomics, proteomics, metabolomics) methods, particularly next-generation sequencing approaches that identify both culturable and non-culturable microbes, play a key role here. These technologies continue to mature, fueling the hope that refined single-cell analytics (Kaster and Sobol 2020; Vollmers et al. 2022), innovative in situ cultivation techniques based on new materials (Zoheir et al. 2022), and the implementation of artificial intelligence for pattern recognition and data analysis (Ardern et al. 2023) will rapidly advance our picture of the complex dynamic processes in microbial communities. While our primary focus should be on comprehending these complex systems as a whole, future advancements may also include integration of modern biotechnological methods like omics, gene editing, and genetically modified organisms (Doley et al. 2020). As technological progress in whole genome editing is rapidly advancing and various multiplex genome editing technologies, including meganucleases, TALENs, and the CRISPR/Cas system, are nowadays almost routinely used for the modification of model organisms (Zhang et al. 2021), even the perspective of tailored optimization of microbial communities seems feasible (Rubin et al. 2022; Venkataraman et al. 2023). These modern technologies, including heterologous expression and metabolic engineering, can be increasingly incorporated, allowing the generation of new and improved products and services, such as plant growth promoters, phytopathogen controllers, and biofactories for production of fuels and pharmacological compounds (Vitorino and Bessa 2017). Beyond its significance for innovations in plant-based agriculture, microbiome engineering is crucial for animal-based agriculture and particularly for integrated circular processes, which will be discussed in the following chapters.

## Animal-based agriculture

**Fig. 1:**
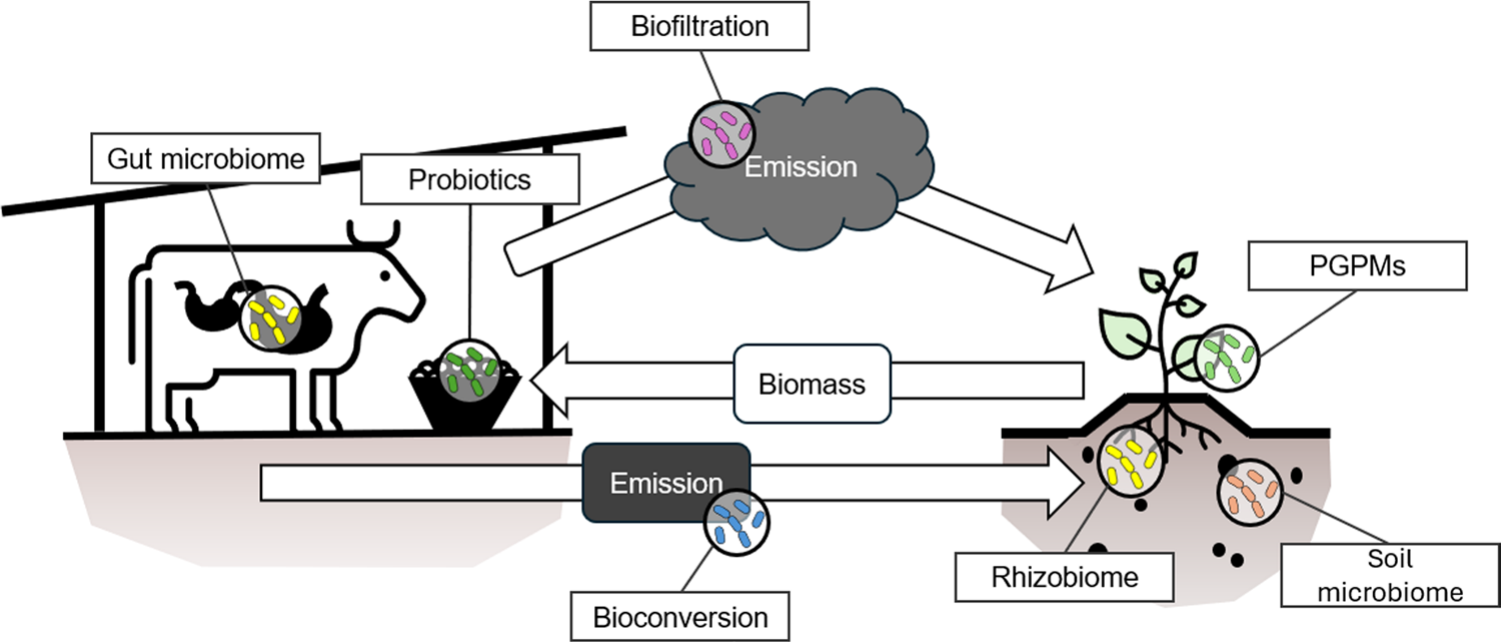
Strategies for utilizing microorganisms in the interaction between animal- and plant-based agriculture include minimizing harmful gaseous emissions through probiotic feed and modifications to the gut microbiome of animals. These emissions can be utilized in plant cultivation via microbial biofiltration, while solid or liquid emissions can be processed through microbial bioconversion. Plant growth is further enhanced by employing plant growth–promoting microorganisms (PGPM) and modifying microbial communities in the rhizosphere and soil to improve plant health and biomass yields. Note that microbial protein is used both as feed and as prebiotics

Animal farming encompasses the raising of livestock and fish for meat, dairy, and eggs, contributing both directly and indirectly to food production. Approximately one-fifth of global caloric intake is based on animal products (Mottet et al. 2017). Due to this demand of animal protein in developing countries, intensive use of antibiotics has become a major problem, resulting in antibiotic residues in animal-derived products, and eventually, antibiotic resistance spread throughout the environment. Antibiotic-resistant genes (ARG) spread in microorganisms therefore pose a great public health concern (Manyi-Loh et al. 2018). The challenges that follow have international dimensions, as there are no geographic boundaries to impede the spread of ARGs. Calls for strengthening of regulations that direct antibiotic manufacture, distribution, dispensing, and prescription are therefore necessary for a sustainable agriculture (Adelowo et al. 2020). Plant and animal agriculture are inherently interconnected, particularly through biomass material flows and related microbial conversion methods, which are increasingly emphasized in the context of a sustainable circular economy (Fig. 1). In animal agriculture, strategies are being developed to reduce harmful gas emissions through the use of probiotic feeds and modifications to the gut microbiome of animals. Additionally, gaseous emissions from animal facilities can be converted via microbial biofiltration, while solid and liquid emissions can be processed through microbial bioconversion for use in crop production. The following sections will explore these strategies in more detail, differentiating between terrestrial livestock (chapter “Livestock-based agriculture”) and marine aquatic animals (chapter “Aquaculture”), which have distinct requirements and possibilities for using microbial tools.

### Livestock-based agriculture

This sector of agriculture, involving the breeding, feeding, and care of livestock such as cattle, sheep, goats, pigs, poultry, and other domestic animals, places a significant strain on the environment globally (Gržinić et al. 2023; Andretta, 2021). For instance, in Germany alone, pigs and poultry are raised on over 200,000 farms, leading to considerable environmental pollution from feces and manure, as well as substantial emissions of CO_2_, NH_3_, and other volatile compounds. The waste generated from animal husbandry can be repurposed into valuable resources through the utilization of biogas plants (Esteves et al. 2019; Abanades et al. 2022). In these facilities, microbial anaerobic digestion (AD) is used as an effective process to convert organic waste into biogas and other valuable products. Again, the abovementioned molecular techniques, especially sequencing-based metagenomic analysis combined with metabolic flux analysis, play a crucial role in gaining a deeper understanding of the microbiome responsible for the AD bioprocesses in such an artificial rumen to enable improvements in process efficiency and the output of specific products (Harirchi et al. 2022; Raja Ram and Nikhil 2022). Interest in biogas, which typically consists of methane (CH_4_), carbon dioxide (CO_2_), and small amounts of impurities such as hydrogen sulfide (H_2_S), nitrogen (N_2_), oxygen (O_2_), and volatile organic compounds (VOCs), has increased significantly as an alternative to natural gas. In recent years, research is driven into the development of specific microorganisms for in carbon capture and utilization (CCU) technologies (Onyeaka and Ekwebelem 2023) as well as membrane processes for biogas purification and upgrading, particularly through the use of polymer and ceramic membranes (Francisco López et al. 2024; Tomczak et al. 2024). A recent technology assessment shows that the global biogas industry has grown by 90% in the last 10 years to 120 GW in 2019 and that further expansion will be significantly influenced by regulatory conditions on exploration, production, processing, environmental impact assessment, marketing, and waste disposal (Abanades et al. 2022).

While biogas technology is promising for the development of sustainable technologies in the livestock sector, it does not solve the problem of gaseous emissions from factory farming. Methane emissions occur during the digestive process of animals, especially ruminants such as cattle and sheep, where methane is produced as a by-product of the digestion of fibers in the rumen, and ammonia is emitted from animal excreta, especially urine, which accumulates and decomposes in stables. One possible solution to this problem lies in influencing the microorganisms responsible for the gaseous emissions. The use of probiotics in animal husbandry, which have a positive effect on health and production, can improve the gut microbiome and strengthen the immunity of both ruminant and non-ruminant animals, which can have a positive effect on overall production performance. Probiotics can also contribute to improving the efficiency of rumen fermentation and reducing methane production in ruminants (Bhogoju and Nahashon 2022; Mahesh et al. 2021). A detailed study based on 16S rRNA amplicon sequencing data shows that the dynamics of the rumen microbiome are determined by stochasticity, which is constrained by deterministic effects of diet and age. As the rumen microbiome evolves from birth to adulthood in cows, animals share a set of core species that colonize early and persist into adulthood (Furman et al. 2020). However, since the dynamics of the late successional taxa strongly depended on the composition of the microbiome in early life stages, it seems likely that an optimal diet can contribute to improved rumen fermentation already in early calfhood. Of the numerous microorganisms that have already been considered as probiotics (tabular overviews can be found in Bhogoju and Nahashon 2022; Mahesh et al. 2021)), some, such as *Bacillus*-based ones, have also been shown to be beneficial in regulating odor gas emissions of industrial facilities (Young and Yun 2019). While the approaches discussed here focus on thermal utilization (biogas) or avoidance (microbiome engineering) of emissions from animal husbandry, new cycle-based methods (discussed below) are concerned with the reuse and upcycling of emissions into higher-value residues.

### Aquaculture

**Fig. 2:**
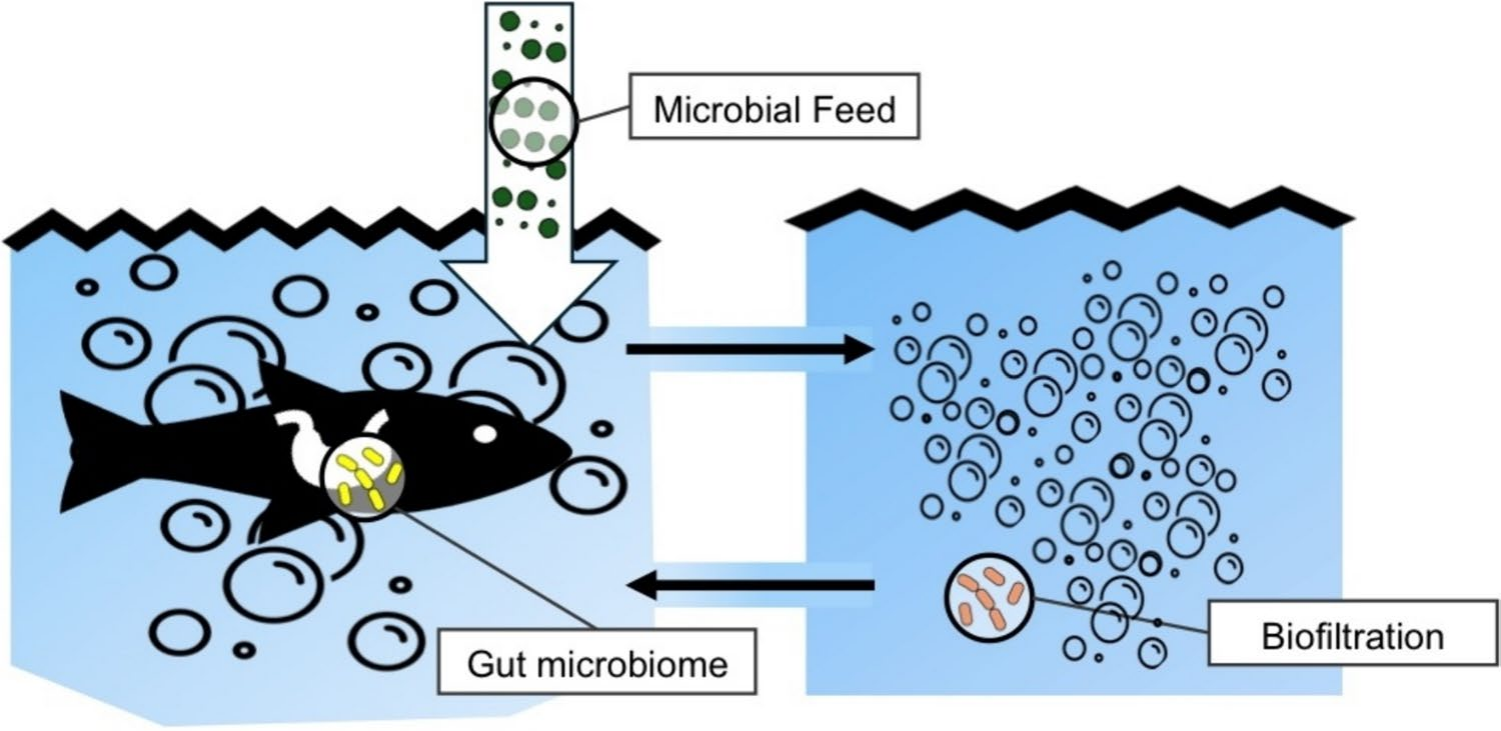
Strategies for using microorganisms in aquaculture include optimizing sustainable feed through microbially produced alternatives, manipulating the gut microbiome to enhance animal health and biomass yields, and extensively employing microbial biofiltration methods to purify recirculating water and minimize resource consumption. This purification process can also be integrated with plant cultivation in systems like aquaponics (Chapter “Towards circular processes—coupling of animal and plant agriculture”)

Aquaculture, with its global production of fish and seafood surpassing 110 million tons in 2018 and involving approximately 425 farmed species (Naylor et al. 2021), is pivotal in animal-based agriculture by significantly contributing to global food production, meeting the increasing protein demand, and ensuring food security. Aquaculture offers several advantages over conventional livestock farming, including more efficient use of space, lower consumption of resources, especially agricultural land, water, and often a lower carbon footprint. Although aquaculture is often seen as a more sustainable and resource-efficient alternative to conventional livestock farming, the management of pathogens, parasites, and pests remains a challenge for sustainability across the industry, and the impact of climate change on aquaculture is considered uncertain and difficult to validate (Naylor et al. 2021). As a result, there is growing pressure on the aquaculture industry to adopt comprehensive sustainability measures (Fig. 2). Current areas of focus include the development of segregated indoor farms, the search for alternatives to fishmeal as supplemental protein feed, and the use of effective microbes to control water quality (Kamalam and Pandey 2022; Manan et al. 2023). Modern indoor farms use recirculating aquaculture systems (RAS), which harbor complex microbial communities that are directly affected by the operation of the system. It has been observed that the operation of freshwater recirculating systems drives bacterial community shifts in the biofilter around a stable nitrifying consortium of ammonia-oxidizing Archaea (AOA) and fully ammonia-oxidizing (comammox) *Nitrospira* (Bartelme et al. 2017; Preena et al. 2021). This suggests that multiple ammonia-oxidizing lifestyles coexist within the nitrifying consortium and contribute to a stable cycling process by reducing nutrient loading. These microorganisms can complete the entire nitrification process independently, which not only challenges the classical two-stage nitrification theory but also updates the long-held view of microbial ecological relationships in the nitrification process (Zhang et al. 2024).

*Teleosts*, the most diverse vertebrate group and an important component of the growing global aquaculture industry, are receiving considerable scientific attention. Recent advances in high-throughput sequencing technologies have enabled research into the gut microbiome of teleosts, particularly in the context of sustainable aquaculture, focusing on topics such as nutrition, immunity, artificial selection, and closed-loop systems. Research on the gut microbiome reveals the impact of aquaculture, highlights key deterministic forces, and has relevance for practical applications related to nutrition and immunity (Perry et al. 2020). For example, methodological and conceptual knowledge gaps can be closed to investigate areas such as feed optimization, vaccination, pro- and prebiotic mechanisms, and the presence and effects of bacteriophages in RAS (Diwan et al. 2024). This approach also enables artificial hologenome selection, where artificial selection is applied not only to the host organism but also to its associated external microbial community (collectively referred to as the “hologenome”) to optimize the symbiotic relationship to improve host health, increase disease resistance, or enhance productivity in agricultural or aquaculture systems. In addition, these methods can be used to investigate basic physiochemical and microbiological properties of water and dysbiosis as biomarkers for aquacultured organisms (Perry et al. 2020).

## Towards circular processes—alternative feeds in animal agriculture

The instances outlined in chapter “Aquaculture” indicate that the growth of aquaculture is propelled by enhancements in process efficiency. There is a concentrated effort on refining operational performance, specifically concerning the nutrition and health of cultivated fish. The increasing trend to achieve natural or organic certification is driving, for example, the investigation of probiotics to promote health by improving the internal microbial balance (El-Saadony et al. 2021). Despite ample evidence of the benefits of microorganisms—including algae, bacteria, fungi, archaea, protozoa, and viruses—their use in nutrition remains limited, particularly in the development of natural medicines and therapies. Similar as in plant and feedstock agriculture, there is a need to investigate in detail the different functions of microorganisms used in feeds to explore their potential (Wan-Mohtar et al. 2022). For example, microalgae, high in fatty acids, essential amino acids, and high value carbohydrates, hold great promise as alternative protein source in aquaculture to replace the current use of around 25% of the world’s fish catch for aquafeed production (Ma and Hu 2024; Tham et al. 2023). Furthermore, although development is still in its infancy, microalgae-based feed is seen as a promising alternative for livestock and poultry production (Kusmayadi et al. 2021; Saadaoui et al. 2021). Since algae metabolize nitrate, phosphorus, and sulfur compounds during growth in addition to light and CO_2_, the integration of microalgae and fish cultivation can also be beneficial for wastewater management in aquaculture (Vijayaram et al. 2024). In a recent study, a photo-biofilter was developed to determine the purification capacity of an immobilized co-culture of the microalgae *Tetradesmus dimorphus* and nitrifying bacteria isolated from a salmon RAS. Sequencing-based analyses showed that the microbial community in the biofilter contained bacteria from the genera *Flavobacterium*, *Microbacterium*, *Raoultella*, *Sphingobacterium*, and *Pseudomonas* and efficiently removed ammonium, nitrate, and phosphate simultaneously in continuous operation (Rodríguez-Leal et al. 2023).

Insects are increasingly being promoted as food and feed worldwide, but their harvest is threatened by overexploitation, habitat change, and pollution, necessitating the development of sustainable harvesting methods. Insect farming, which can be carried out both on small farms and in large industrial facilities, is ecologically beneficial compared to livestock farming as it requires less land and water, emits fewer greenhouse gases, enables high feed conversion, can convert low-value organic by-products into valuable food or feed, and can be used as animal or aqua feed to replace increasingly scarce and expensive fishmeal, for example (Hawkey et al. 2021; Mastoraki et al. 2020). Agricultural insect species destined for production must be rigorously assessed for potential risks to human, animal, plant, and biodiversity health (Aidoo et al. 2023; van Huis and Oonincx 2017). The black soldier fly (*Hermetia illucens*) is a subtropical dipteran species native to the Americas. Due to their high ability to convert biological waste into insect protein and fat, black soldier fly larvae (BSFL) are widely used in insect factories to produce sustainable, high-quality feed ingredients (Abd El-Hack et al. 2020).

A recent study investigated how the feed substrate and gut microbiome contribute to protein and fat synthesis BSFL to ensure optimal larval development. The results indicated that larvae fed with high-quality feed (chicken feed) had a higher fat biomass, while those fed with medium-quality feed (wheat bran) had a higher protein biomass. The initial nutrient content alone could not fully explain the growth and nutrient utilization of the larvae, but the microbial metabolism in the gut of the BSFL played a crucial role. Chicken feed improved fatty acid metabolism in the midgut and thus promoted fat synthesis, while wheat bran stimulated amino acid metabolism in the larval gut, and thus improved protein synthesis. These results emphasize the importance of the function of the gut microbiome and show that, influenced by the type of diet, it is crucial for efficient conversion of organic waste into high-quality insect protein and lipid (Li et al. 2024). This result aligns with findings from typical composting processes, in particular those involving food waste, which offer abundant organic material for conversion into fertilizer, thereby enhancing soil quality (Mahish et al. 2024). Insects are increasingly being used to upcycle liquid- and solid-waste materials such as manure and feces. This process can be applied in agriculture and waste management, where insects, especially BSFL, can convert organic waste into high-value proteins and fats (Cammack et al. 2021; Rossi et al. 2023). Despite their potential, these processes often encounter legal restrictions. Many countries have stringent regulations and guidelines, especially concerning the use of insect products as animal feed or even for human consumption. Due to these regulatory constraints hindering the adoption and expansion of insect upcycling practices, thorough studies are necessary to ensure that insect products meet health and environmental standards by being free from contaminants and pathogens (Siddiqui et al. 2024). However, it should be noted that the production of microbial protein feed from waste provides stable and high-quality proteins that are used for aquaculture in China, for example (Zhou et al. 2023). In addition, Calysta should be mentioned as one of the leading companies whose process based on methanotrophic microorganisms has already reached a high technology readiness level (TLR) (Gęsicka et al. 2021).

## Towards circular processes—coupling of animal and plant agriculture

As discussed in the context of Fig. 1, the coupling of animal and plant agriculture has been used since ancient times as a symbiotic relationship for sustainable food production, using organic matter in the form of manure to promote soil fertility and thus plant growth. In turn, arable farming provides feed for livestock, completing a nutrient cycle that maximizes resource efficiency, increases biodiversity and the resilience of agroecosystems, and reduces reliance on synthetic fertilizers and pesticides. Innovative agricultural practices such as rotational grazing, agroforestry, silvopasture, and similar approaches where crops are grown and livestock grazed simultaneously on the same land, serve to holistically increase productivity while maintaining environmental integrity. It is crucial to emphasize that such innovations are essential for transforming a significant portion of agriculture towards more sustainable practices. Many of the microorganism-based approaches discussed above are already in, or even beyond, the testing phase, aiming to make conventional large-scale agriculture more ecological and sustainable. This is particularly true for the use of microorganisms in soil improvement (chapter “Plant-based agriculture” and chapter “Microbiome engineering”), as substitutes for harmful agrochemicals (chapter “Plant-based agriculture”) or antibiotics in animal husbandry (chapter “Animal-based agriculture”), as (probiotic) additives in animal nutrition (chapter “Livestock-based agriculture” and chapter “Alternative feeds in animal agriculture”) and aquaculture (chapter “Aquaculture”), and as active components in biogas technology (chapter “Livestock-based agriculture”). Nevertheless, since much of the conventional agriculture methods remain tied to extensive land use, and with climate change leading to a decrease in available arable land, novel strategies must be devised to safeguard the future food provision for the expanding global population.

Closed-loop systems that enable the highly efficient use of available land and raw materials for agricultural production processes would be ideal. Controlled environment agriculture (CEA) in urban areas is one such approach that addresses future needs (Dsouza et al. 2021; Engler and Krarti 2021; Marvin et al. 2023). Such urban CEA systems include, in particular, cultivation techniques such as climate-controlled greenhouses and plant factories with artificial lighting, nowadays even animal factories (The Guardian 2022), which can deliver high production yields regardless of external environmental conditions. For example, a recent study suggests that under optimized conditions, vertical indoor farming could deliver up to 600 times the current global average annual yield of wheat (Asseng et al. 2020). Recent research indicates a growing recognition, acceptance, and anticipation of the evolution of “agritecture” (the fusion of agriculture and architecture) as a pivotal mechanism for reshaping and modernizing future urban food systems, notably across Asia (Ebissa et al. 2024; Zhou et al. 2022).

Closed-loop systems for coupled animal and plant-based CEA can be realized through aquaponics (Fig. 2). This is a sustainable cultivation method that combines aquaculture with hydroponics (the cultivation of plants in water). In an aquaponics system, fish excrement provides nutrients for the plants, and the plants in turn filter and purify the water for the fish (Baganz et al. 2022; Kushwaha et al. 2023; Rufí-Salís et al. 2020; Wirza and Nazir 2021). In a closed system, water consumption and the use of chemical fertilizers are therefore minimized to enable the simultaneous production of fish and vegetables in an environmentally friendly way in a controlled environment. Similar as outlined above for conventional agriculture, the significance of plant growth–promoting microorganisms in CEA is increasingly recognized for enhancing plant growth and disease resilience. In aquaponics, where plants and fish coexist, microbial processes are vital for nutrient recycling, mirroring nutrient interactions found in traditional systems. Yet, managing microbial competition, particularly for micronutrients like iron, poses challenges, necessitating supplementary measures that impact system sustainability and profitability (Bartelme et al. 2018). Understanding the complex bacterial populations in aquaponics systems facilitates the development of sustainable and healthy food production systems. The aquaponics microbiome, with its diverse bacterial composition in the different system compartments, emphasizes the importance of establishing these ecosystems for optimal system function. Here, bacteria dominated by *Proteobacteria* and *Bacteroidetes* at the phylum level play a fundamental role and human pathogens in aquaponics products can be avoided through appropriate hygiene measures (Dinev et al. 2023; Kasozi et al. 2021).

## Closing the loop—sustainable and resilient systems for future agriculture

Exploring the potential of different trophic levels including terrestrial and aquatic livestock, plants, insects, and microorganisms, we will delve into the role of microalgae within closed CEA systems to envision scenarios for resilient- and resource-efficient agriculture. As previously mentioned, intensive livestock farming generates liquid and solid waste (manure, feces) as well as gaseous emissions and metabolic waste heat, which could potentially be utilized for other production processes. Utilizing the gaseous and energetic emissions from livestock stables for microalgae cultivation thus presents a logical approach. Microalgae and other photosynthetic microorganisms (PMO) can upgrade the CO_2_ exhaled by the animals into valuable substances and are also capable of filtering nitrogen and sulfur compounds or other gaseous or aerosol-bound substances from the air. Therefore, it is not surprising that the cultivation of PMO has become an established technology for producing valuable biomass in a resource-efficient manner (Fernandes et al. 2015). The selection of PMO is not limited to naturally occurring strains; with the aid of genetic engineering, recombinant species can also be engineered to produce specific biogenic compounds in high yields, enabling the economical production of high-value products for energy, food, or health applications, including enzymes or therapeutic antibodies (Ahmad et al. 2020; Udayan et al. 2021; Udaypal et al. 2024).

PMO and other microorganisms are employed in bioremediation to eliminate pollutants from liquid, solid, and gaseous waste streams (Bala et al. 2022; Roy et al. 2022; Touliabah et al. 2022). For instance, microorganisms on solid support materials serve as potent biofilters for air purification, removing air pollutants from exhaust gases (Barbusiński et al. 2021; Hussain et al. 2021). PMO are also utilized in the bioremediation of liquid media, such as improving nitrogen waste management in recirculating aquaculture systems (Ramli et al. 2020). While PMO’s use for CO_2_ sequestration is well-established (Cheah et al. 2015; Kumar et al. 2011; Onyeaka et al. 2021), its potential for reducing gas emissions from animal housing remains is not well studied. While model tests in the laboratory suggested the feasibility of this approach (Kang and Wen 2015), it was only recently possible to experimentally investigate the direct coupling of an algae reactor with a chicken coop (Glockow et al. 2023). To this end, a cone-shaped, helical tube photobioreactor was utilized for the continuous cultivation of *Arthrospira* spec. (*Spirulina*) by using the exhaust air from a chicken coop. Measurement of CO_2_ and NH_3_ concentrations demonstrated that the algae reactor efficiently purified the air for several weeks while generating algae biomass with high efficiency. Genomic characterization of the *Spirulina* cultures offered insights into the dynamics and metabolic processes of the microbial community. This shows that the production of value-added biomass by sequestering gaseous emissions from livestock barns is possible and represents an important piece of the puzzle for future circular agricultural CEA systems.

**Fig. 3:**
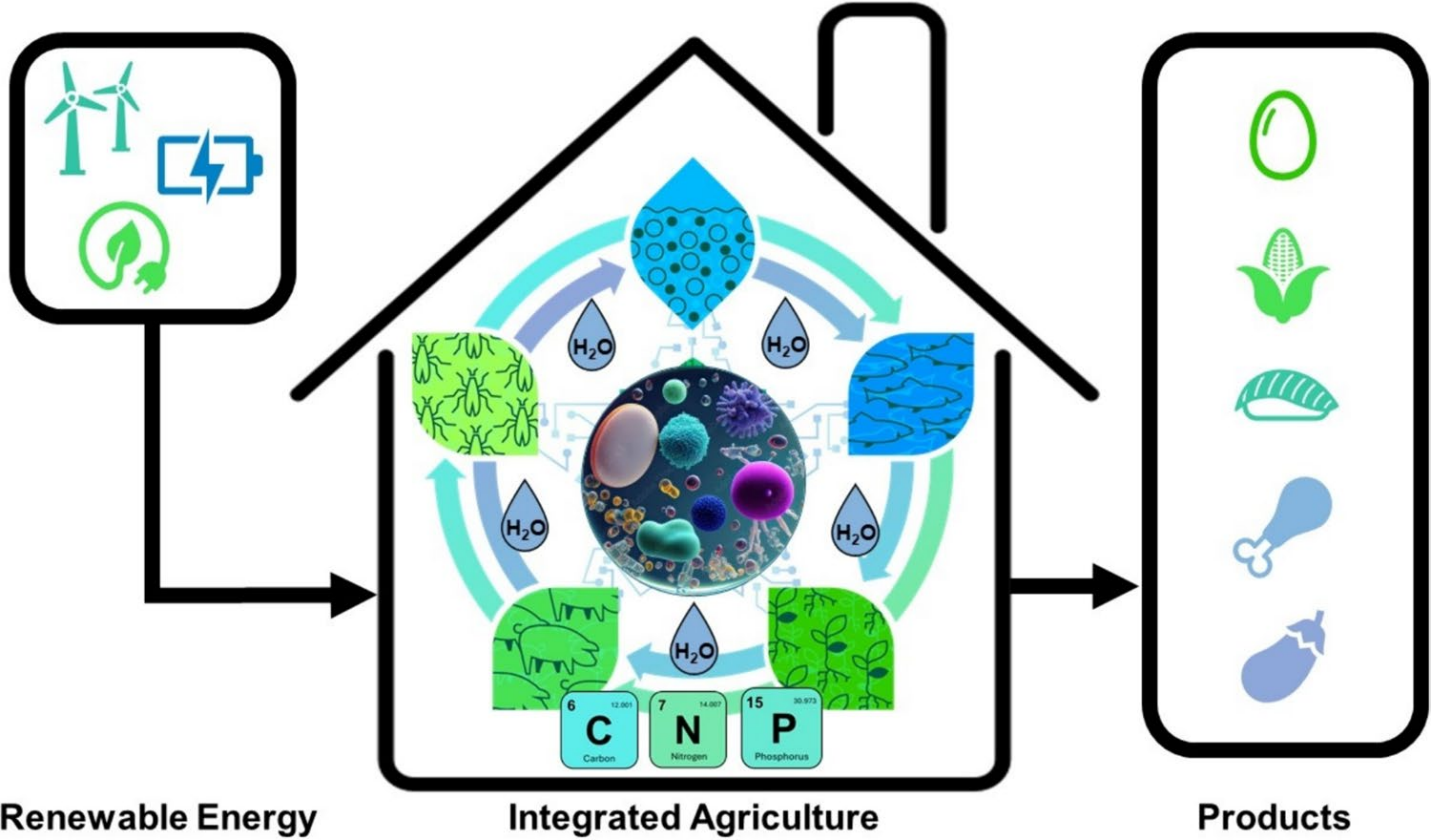
Closed-loop system for coupled animal and plant-based agriculture in a controlled environment. The solid, liquid, and gaseous emissions from livestock are upgraded by microalgae and insects to higher value biomass in order to produce feed, e.g., for aquaculture. This is combined with vegetable farming according to the principle of aquaponics to produce food for humans and at the same time residual materials for animal feed. The recirculation system is powered by renewable energy and enables an optimal reuse of nitrogen, phosphorus, micronutrients (turquois), and, above all, water (blue), while minimizing environmentally harmful emissions. The microorganisms essential for the operation and coupling of the trophic levels play a key role in this system

The aspects of the current state of industrial agriculture discussed in this and the previous chapters suggest that, from a technical point of view, closed-loop systems for coupled animal and plant-based agriculture should be possible in a controlled environment. A possible realization is shown schematically in Fig. 3. Here, the solid, liquid, and gaseous emissions from livestock are upgraded to higher value biomass by microalgae and insects in order to produce feed for aquatic and terrestrial livestock. Integrated aquaculture not only produces food but also waste products, which are used as fertilizer for vegetable farming according to the principle of aquaponics, in order to produce food for humans on the one hand and waste products for animal feed on the other. The recirculation system is powered by renewable energy and enables the maximum reuse of nitrogen, phosphorus, micronutrients, and above all, water, while minimizing environmentally harmful emissions. The microorganisms essential for the operation and coupling of the trophic levels play a key role in this system. The explanations in this mini-review clearly show the evidence that such a circulation system is only possible in interaction with the associated microbial communities. As nature impressively proves that such complex ecosystems work, there is hope that we are able to develop technical systems to mimic closed productive ecosystems and utilize them for the production of healthy sustainably produced food.

It should be noted that it is obvious that breeding animals as a source of food is not only ethically but also ecologically questionable due to the high consumption of resources. For this reason, there are extensive efforts to develop animal-free foods. As an example, reference should be made not only to the aforementioned use of edible PMO for foods (Linder 2019), but also to the efforts to produce human food directly by using microbial techniques. The latter approach is considered as microbial food revolution (Graham and Ledesma-Amaro 2023), and focuses on the emergence and use of new tools, particularly in synthetic biology, to expand the uses of microorganisms to meet our nutritional needs. This approach addresses both the use of microbes to produce whole foods from their biomass and the use of cell factories to produce starting and intermediate products as well as highly functional and nutritional ingredients. Although this approach is rapidly gaining momentum, given the enormous economic importance of industrial livestock farming, we believe that it makes both economic and environmental sense to develop new approaches in the traditional animal-based agriculture.

## Conclusions and outlook

The increasing global population and the looming challenge of climate change are compelling us to develop fresh innovations for a sustainable overhaul of traditional agriculture. While microbial communities have been utilized since ancient times, their specific application for transforming previously neglected residual and waste streams, enhancing plant and animal well-being, and generating alternative food and feed sources is only now gaining significant momentum. This advancement primarily stems from the groundbreaking biological methodologies of the past two decades, notably deep sequencing and omics-based techniques, which are additionally propelled by the ongoing surge in machine learning and artificial intelligence (AI). Coupled with advanced analytics at the individual cell level and pioneering in situ cultivation methods employing novel materials, this technological advancement will rapidly progress and furnish us with a deeper comprehension of the composition, dynamics, and utility of intricate microbiomes, predominantly comprised of “microbial dark matter” (MDM), encompassing microbes that defy cultivation in laboratory settings. Moreover, delving into agriculturally pertinent holobiomes will yield insights into novel microbes, metabolic pathways, and enzyme catalysts, thereby facilitating novel applications in industrial biotechnology, such as food production, odorants, and medically valuable molecules.

The continuous progress in technology is also empowering us to address the pressing need for agricultural transformation. This includes the research and development of more efficient methods for generating and storing renewable energy to enable economic solutions for the ecologically sustainable production of food in closed entities. In the future, closed-loop processes will be made available by AI-supported devices currently under development for robotic execution and data-based monitoring of trophic levels in order to combine maximum efficiency with production safety in the most closed cycles possible. Based on our analysis, which reveals that all the essential components are already set to reshape traditional agriculture towards sustainability, we conclude by echoing the words of the German philosopher Johann Wolfgang von Goethe (1749–1832): “Knowing is not enough; we must apply. Willing is not enough; we must do.”
